# Nasolacrimal duct obstruction following allo-HSCT: an overlooked manifestation of ocular graft-versus-host disease

**DOI:** 10.1038/s41433-026-04776-8

**Published:** 2026-08-01

**Authors:** Kangkang Xu, Juanjuan Hu, Wan Li

**Affiliations:** 1 Department of Ophthalmology, Union Hospital, Tongji Medical College, Huazhong University of Science and Technology, Wuhan, China; 2 Department of Operating Room, Union Hospital, Tongji Medical College, Huazhong University of Science and Technology, Wuhan, China

**Keywords:** Lacrimal apparatus diseases, Haematological cancer, Immunopathogenesis, Immunosuppression, Conjunctival diseases

Allogeneic hematopoietic stem cell transplantation (allo-HSCT) is a curative treatment for a broad spectrum of benign and malignant haematological disorders [1]. However, graft-versus-host disease (GVHD) remains a major long-term complication, with ocular involvement occurring in 40–60% of recipients [2]. Ocular GVHD typically affects the ocular surface system, and while it rarely causes permanent vision loss, it substantially impairs patients’ long-term quality of life. Nasolacrimal duct obstruction (NLDO) is an underrecognised manifestation of ocular GVHD, with only two isolated case reports in existing literature [3, 4]. Evidence regarding the safety and efficacy of surgical management for NLDO in this immunocompromised population remains scarce, limiting standardised clinical decision-making.

We retrospectively analysed three patients who developed delayed NLDO after allo-HSCT at our centre. All cases were confirmed by lacrimal irrigation and computed tomography-dacryocystography, and all received endoscopic dacryocystorhinostomy (enDCR) with bicanalicular silicone intubation.

The case series comprised two female and one male patient, with underlying diagnoses of aplastic anaemia, acute myeloid leukaemia, and primary myelofibrosis, respectively. Symptom onset occurred 1–4 years after transplantation, consistent with the progressive phase of chronic GVHD. One patient had bilateral obstruction, and two had unilateral obstruction; two presented with chronic dacryocystitis with persistent purulent discharge, while one had isolated epiphora without infection. One patient had a prior confirmed diagnosis of ocular GVHD-related dry eye. All procedures were completed without intraoperative or perioperative complications, and silicone intubations were removed 3 months postoperatively. At follow-ups ranging from 1 to 6 months, all patients achieved complete resolution of presenting symptoms, with lacrimal irrigation confirmed at each visit. Notably, the patient with pre-existing dry eye experienced significant symptom exacerbation 6 months after surgery, which did not respond to conventional lubricants and ultimately required permanent punctal plug insertion.

The delayed clinical presentation supports a chronic inflammatory pathogenesis. We hypothesise that donor T cells directly attack the nasolacrimal duct mucosa, triggering persistent inflammation and progressive submucosal fibrosis [4]. In addition, cicatricial conjunctivitis, a common feature of chronic ocular GVHD, may induce ascending inflammation along the lacrimal drainage system, further contributing to ductal stenosis [2].

The clinical outcomes after enDCR and external dacryocystorhinostomy were comparable [5]. Compared with conventional external dacryocystorhinostomy, enDCR minimises soft tissue trauma and reduces perioperative infection and bleeding risks, making it well suited for immunocompromised transplant recipients. We also noticed that NLDO may provide “passive protection” for the ocular surface by reducing tear loss. Surgical relief of obstruction can therefore unmask latent dry eye or worsen pre-existing disease. This effect may also explain why NLDO is underrecognised in GVHD patients, as reduced tear production often masks epiphora symptoms until secondary dacryocystitis develops. Comprehensive preoperative dry eye assessment is thus essential, and concurrent punctal plug placement may be considered for high-risk patients, with long-term postoperative ocular surface monitoring.

In conclusion, NLDO is an overlooked manifestation of chronic ocular GVHD after allo-HSCT. EnDCR is safe and effective for these patients, and careful perioperative evaluation and management of dry eye are critical to optimise long-term patient outcomes.

## Data Availability

Data sharing not applicable to this article as no datasets were generated or analysed during the current study.
